# Higher Charges for Distant Patients?

**Published:** 1921-09-03

**Authors:** Hedley Lucas

**Affiliations:** House Governor and Secretary. The Queen's Hospital, Birmingham.


*To the Editor of* The Hospital.

### "Higher Charges for Distant Patients?"

*To the Editor of* The Hospital.

Sir,—I welcome the opportunity of dealing with the points raised in your note of this week respecting the "Birmingham Queen's Hospital" scheme for payment by patients residing in districts outside Greater Birmingham.

A letter which is sent to all such patients when notified for admission is self-explanatory, explicitly stating that anyone who is unable to pay three guineas per week should produce to the undersigned satisfactory evidence of such inability. No case is refused admission if unable to defray such cost, exemption or reduction being granted after investigation. From May 1, 1921, the date upon which the scheme was launched, to August 15, 1921, thirty-four of the 125 patients coming within its scope have stayed in the hospital longer than three weeks—*en passant* the "Queen's" is a medical school.

The purpose of my Committee in limiting the charge to £10 10s. for the whole stay (*i.e.*, where it exceeds three weeks, and the patient is able to pay) is to cover protracted cases, and the fact that during the above-mentioned three and a-half months almost one-third of the patients come within that category has justified the Committee's assumption and action. It is not that "the Committee feels that the charge is too high and is anxious to make it appear lower," but that a reasonable limit is set in respect of long cases.

The policy is certainly not "to discourage patients coming from a distance," nor has it had that effect. The scheme has been inaugurated because it is considered that patients who receive the special treatment which is afforded by the "Queen's," which is not only a general hospital but a medical school, and serves a wide area (from not a few districts of which little, if any, pecuniary support is forthcoming, and in which, be it noted, voluntary hospitals are situated), should financially assist the Queen's Hospital, especially as it has a large deficit.

The scheme was not in any sense intended to be, nor can it be construed as being rigid—on the contrary, as indicated at the outset of my letter, it is undoubtedly flexible; moreover, it is working well.

May I say that your use of the comparative adjective "higher" in the penultimate [sentence of your] paragraph suggests that patients other than those to whom the scheme applies are charged where able to pay—that is not so?—Yours faithfully, Hedley Lucas,
House Governor and Secretary.

The Queen's Hospital, Birmingham.
August 20, 1921.

[We are glad to publish Mr. Lucas's courteous letter, but are still not clear why the scheme applies only to patients living at a distance, unless one of its objects is to encourage them to seek treatment at hospitals nearer than the Queen's to their own homes.—Ed. The Hospital.]

